# Nurses’ experience of nasogastric tube feeding under restraint for Anorexia Nervosa in a psychiatric hospital

**DOI:** 10.1186/s12910-024-01108-x

**Published:** 2024-10-10

**Authors:** Berit Støre Brinchmann, Mette Spliid Ludvigsen, Tove Godskesen

**Affiliations:** 1 Faculty of Nursing and Health Sciences, Nord University, Postboks 1490, 8049 Bodø Norway; 2 Regional Centre for Eating Disorders, Nordland Hospital Trust, Bodø, Norway; 3 Department of Clinical Medicine-Randers Regional Hospital, Aarhus University, Aarhus, Denmark; 4 Centre for Research Ethics & Bioethics, Department of Public Health and Caring Sciences, Uppsala University, Uppsala, Sweden

**Keywords:** Anorexia nervosa, Coercion, Ethics, Involuntary treatment, Nasogastric feeding, Nursing, Restraint, Physical, Social Vulnerability.

## Abstract

**Background:**

Anorexia nervosa is a complex mental disorder that has severe physical and psychological consequences, often requiring hospitalisation, and in the most severe cases, patients receive coercive treatment. Among the various nursing tasks associated with encountering these patients, the administration of nasogastric tube feeding under restraint stands out. It is crucial to recognise and address the unique practical and ethical challenges nurses face when caring for adults struggling with severe anorexia nervosa. The aim of the study was to gain a deeper understanding of registered nurses’ experience of nasogastric tube feeding under restraint in hospitalised patients with severe anorexia nervosa.

**Methods:**

A naturalistic design guided this study. Narrative interview data were analysed using reflexive thematic analysis. The participants were twelve registered nurses recruited from an inpatient ward for adult patients with an eating disorder in a Norwegian psychiatric hospital.

**Results:**

Three main themes were developed: providing good nursing care during coercive treatment; having ethical concerns about nasogastric tube feeding under restraint when the patient reaches a body mass index that is not immediately life-threatening; and having concerns about involving personnel from another ward in the nasogastric tube feeding under restraint procedure.

**Conclusions:**

Nurses find nasogastric tube feeding under restraint to be part of life-saving nursing for patients with severe anorexia nervosa. It raises ethical concerns, especially with patients with a body mass index that is no longer life-threatening. Our results demonstrate the vulnerability of nurses as well as the difficulties and ethical dilemmas of nursing during nasogastric tube feeding under restraint.

## Background

Anorexia nervosa (AN) is a complex mental disorder that leads to severe physical and psychological consequences, significantly reducing quality of life. AN frequently results in extreme malnutrition and a range of associated medical complications. It is associated with a high mortality carrying a five or more times increased risk of death [1].

The most severe AN is a critical condition that can be life-threatening and often requires hospitalisation. The most acutely affected patients may require treatment under restraint to ensure their safety and facilitate recovery. This measure is often taken to prevent self-harm and manage the medical complexities associated with the disease. The presentation of symptoms represents a multifaceted challenge for healthcare professionals due to the interplay of psychological, physiological, and sociocultural factors [2–4]. Despite advances in psychotherapeutic and pharmacological interventions, some individuals with AN find difficulty in engaging with voluntary treatment as they may lack the motivation to change, or refuse to accept, that they have a treatment need [5].

When individuals are unwilling or unable to engage in voluntary treatment, compulsory measures such as nasogastric tube feeding under restraint (hereafter referred to as NGT-FR) become necessary to address life-threatening malnutrition and its consequences.

NGT-FR is a lifesaving intervention in cases of extreme malnutrition due to AN. However, the psychological impact of compulsory measures on the therapeutic alliance between nurses, clinicians and patients should also be carefully considered. A recent metasynthesis review indicates that manual physical restraint is, generally, unpleasant for nurses and healthcare staff but necessary as maintaining life is always paramount [3]. Nurses in a recent study maintain that they do not feel that physical restraint damages the therapeutic relationship, but that the support of the team is important [6]. A qualitative study of nursing assistants using NGT-FR shows that they commonly experienced emotional distress, physical exhaustion, physical injury and aggression as a result of their manual restraint use [7]. These findings provide some insight into how healthcare professionals understand physical restraint. NGT-FR raises both legal and ethical considerations about patient vulnerability, autonomy, integrity, and potential harm [4, 9–11]. In cases of AN, where patient insight is often impaired, nurses must strike a delicate balance between recognising individual vulnerability, respecting patient autonomy, and preventing harm associated with severe malnutrition and death [11, 12]. NGT-FR as a means of coercive treatment sparks discussion among nurses about the acceptable limits of medical intervention and the ethical implications of overriding a patient’s refusal of treatment [11, 14–17]. Considering the expected increase in the incidence of AN and the high mortality, it is important to address the challenges encountered by nurses when working with adults with this life-threatening condition [1].

### Norwegian legislation

In recent years, political directives and legislation in Norway have emphasised reducing the use of coercion and enhancing patients’ autonomy. All formal coercive measures such as for example involuntary commitment and involuntary tube or medications must be legally justified. The criteria for formal coercive treatment are as follows: 1) treatment options have been previously attempted and exhausted; 2) serious mental illness; 3) an assessed incapacity of the patient to consent; 4) if there is a danger to the patient’s own or others’ lives, or the prospects of recovery or significant improvement are significantly reduced, or if the condition worsens (§ 3–3). If there is a danger to the patient’s own life or others’, the patient’s consent is not required. The Supreme Court has determined that, legally speaking, eating disorders may constitute serious mental illness (RT. 2015 p. 913). The most commonly used means of coercion in severe AN are the establishment and management of forced treatment (§ 3–3), and the adoption and practice of nutrition without consent (§ 4-4b) [9, 10]. The use of coercion in connection with admission therefore raises legal as well as ethical questions.

## Method

### Aim

The aim of the study was to gain a deeper understanding of registered nurses’ experience of NGT-FR in hospitalised patients with severe AN.

### Design

A naturalistic design informed the trajectory of our study [17]. In alignment with Braun and Clarke’s perspective, drawing upon Gough and Madill’s definition of qualitative research as a creative, reflexive, and subjective endeavour, we view researcher subjectivity as a valuable resource rather than an obstacle to knowledge generation [18, 19]. We believe that the construction of meaning is tied to context, positionality, and situation. To delve into the narratives of nurses who have encountered challenges associated with NGT-FR for hospitalised patients with severe AN, we employed a reflexive thematic analysis approach. Our analytical exploration of the narrative interviews adhered to a naturalistic, inductive, and “data-driven” approach. This ensured that it remained purely reflective of the data content, untethered from any preconceived theories or conceptual frameworks [20].

### Setting

This study focuses on nurses’ experiences with the most severely sick patients with AN under coercive treatment at an inpatient ward providing 24-hour care for adult patients with a severe eating disorder in a Norwegian psychiatric hospital. There is no consensus on the definition of severe AN [21]. The ward in this study followed the diagnostic criteria for severe AN in line with the Diagnostic and Statistical Manual of Mental Disorders, Version Five (DSM-V): body mass index (BMI) < 15; Intentional caloric restriction resulting in weight loss; intense fear of gaining weight; and body image distortion (i.e. believing themselves to be extremely fat, when they are actually normal - or even underweight). In addition, the cognitive consequences of being severely underweight impact the capacity to consent.

The unit has eight beds, and approximately fourteen nursing positions, plus treatment staff. The patient group consisted of individuals receiving both voluntary and involuntary treatment. The patients were mainly women, most aged between eighteen and twenty-five, some in their thirties and forties. Some hospitalised for up to a year, some for a few weeks.

### Recruitment and characteristics of participants

Information about the study was given during a staff meeting, and those wishing to participate contacted the first author. The participant nurses comprised ten women and two men, between thirty-eight and seventy years old and with between two- and nineteen-years’ experience on the inpatient ward. Seven were specialised in psychiatric nursing/mental health care, others in intensive care, diabetes, management, paediatrics, or public health. All had extensive work experience with other patient groups, within both somatic and mental health.

### Data collection

After obtaining informed consent from the participating nurses, the first author conducted the interviews at the hospital in 2022 using an interview guide (Table 1). The interviews lasted 54 min on average. All were audio recorded and transcribed verbatim.

**Table 1:** Interview guide

Interview guide
Can you please share some experiences you have as a nurse for patients with a severe eating disorder.
Can you describe situations where you felt you provided good nursing care – as concretely as possible.
Please describe a situation where you experienced an ethical dilemma.
What are your thoughts on the use of coercion?
What is particularly difficult or challenging when working with this patient group?

### Data analysis

Each author meticulously and iteratively reviewed all the interviews independently and collectively. Through examination, it became evident that many of the participants expressed emotional and deeply personal experiences related to NGT-FR for individuals with severe AN. It became apparent that NGT-FR held considerable significance for them, and we decided to delve into this in the further analysis. We extracted relevant passages from each interview. We extracted and coded the text with manifest meaning, developing an initial understanding of possible thematisation. Reading across the codes, we uncovered latent meanings, facilitating the development of themes. Our final interpretation of meaning was thus derived from comprehensive analysis across all interviews [20].

## Findings

### A narrative about nursing and NGT feeding under restraint

*The patients who stand out most are those who have to be restrained*,* those who struggle physically. In our unit*,* we use staff from another unit to come and take control of these patients*,* and we insert the tube only after they have been given all possible other choices and have been unable to take them. So*,* we hold them still*,* put in the tube and feed them through it. The reason we make use*,* in this way*,* of unknown others*,* even in the knowledge that it may be extra unpleasant for a patient with a history - often young girls – is because we think…we think so profoundly that it’s us who’ll be there day and night. We’ll take care of them after this has happened*,* this session or whatever you call it which is so invasive. I can’t think of any more invasive treatment than inserting a tube in their nose and force feeding them.*

*I’ve sometimes thought that it’d be best if it was me who restrained the patient*,* and that I went in afterwards and asked her how it went*,* whether it had been OK. What exactly had happened. She had been fed*,* and the restraining of her had been only that that seemed necessary to me. I’d held her head in my arm kind of firmly*,* but not too hard. She knew it was me*,* so she didn’t struggle too much. And then I’m holding one hand*,* and someone’s holding the other*,* and there was a third person there while I guided the tube down.*

*What strikes me is the number of hats one has to wear as a psychiatric nurse in this type of unit. In these situations*,* it’s me who talks to the patients before mealtimes. About what they’ll have and agree about what they should eat and what they should do. And then I go in and sit and work with them during the meal. And sometimes it doesn’t work out*,* and then you can either offer a substitute or it has to be the tube. So*,* then I have the role of supervisor during the meal. “You know the consequences; I don’t want you to have to be force fed. I want the best for you. I think the best thing for you is that you eat.” So*,* that’s sort of my role before and during the meal*,* it’s completely different than when we’ve to decide on the use of the tube. Then*,* I have to go in and be somewhat different. I don’t become strict or angry*,* but there’s no longer any place for pleasantries. OK*,* it’s like this*,* now we’ve decided that we’ve tried everything we can. And then I go and call or I go and arrange with the doctor so that we can begin the procedure. We have to finish the meal. And then we come to inserting the tube itself.*

*Some nurses say that you have to just do it. You can’t just keep on trying. But it’s entirely natural for me to continue whilst I’m standing there with the tube and holding them and saying: “You can still drink that nutritional drink; you can still do it.” And*,* sometimes*,* I’m aware that I spend maybe more time than I should. I give them every chance. But then the whole situation is prolonged*,* instead of just saying: “OK*,* now we’ve decided on this.” So*,* then I’m conflicted in myself*,* knowing that this is a fearsome intervention. During the insertion of the tube one is*,* of course*,* a nurse and follow a set procedure as well as possible. And*,* by and large*,* it goes well.*

*When it’s done*,* then I have to look after this patient who I’ve been involved with and seen being tube fed. And I wonder if it’s a good thing that I’ve done all these things*,* that I’ve planned the meal*,* attended the meal*,* decided that there must be tube feeding*,* inserted the tube*,* and then taken care of her after the force feeding*,* or at least tried to.*

*My way of thinking is that I deliver the best care if I’m there the whole time*,* if I follow the patient the whole way…. “You know that I haven’t missed anything*,* there’s nothing that happened to you that’s passed me by. I was there*,* I did it.“*.

*Or should I suppose that the best care would have been perhaps if I had withdrawn*,* or that we had a different system that made them come from another unit to restrain her*,* and that I went out during the procedure. I think about this a lot*,* that it’s one of the most terrible things that I do to a young girl. It gets me every time. At the same time*,* it is care*,* using the tube and forced feeding is care. Nutrition is care*,* so we are delivering care when we do that which is most freedom denying for the patient.*

*But it is this care….my experience of it is….it’s me who’s going to …why do I feel so strongly that I must put on my other hat and go into her room? I feel certain that she knows that I wish her no harm. She knows I don’t want to see her hurt*,* I don’t want to hurt her*,* and I ask: “Is it OK with you that I’m here now? I know that I… we were just in a situation where I fed you against your will.” And then she maybe says: “Yes*,* it’s OK.” So then*,* I sit down – but not too close. As a man*,* I have a certain… yes*,* I have to keep a certain distance. It’s not as though I go straight in and hold her*,* that sort of hugging. But I sit down and try to give a kind of hugs - cognitive hugs – and say: " I care about what just happened*,* and I hope you’ll tell me if there’s anything I could’ve done differently.” A kind of debriefing with the patient – I think it’s needed. And maybe it’s for my own sake*,* maybe it is. But there are things I feel so strongly about that that’s how I want it to be*,* that’s how I’d want to be treated if it were me* (Thomas).

We developed tree main themes: providing good nursing care during coercive treatment; having ethical concerns about continued NGT-FR when the patient reaches a BMI not immediately life-threatening; and having concerns about involving personnel from another ward in the NGT-FR procedure.

### Providing good nursing care during coercive treatment

In patients with severe AN, who have reached a critical point where the situation poses a significant threat to their lives, NGT-FR has to be administered based on medial assessment and legislation.

*Some of these patients seem to have a resistance to care. And it’s not just the care we provide*,* but also their way of taking care of themselves that’s totally failing* (Rosie).

Confronted with the complexities of these trying circumstances, nurses recognise that *the best form of care is (sometimes) compulsory when it’s necessary* (Beatrice). Gina said:

*The compulsory care we give should be so good that when we end it*,* they will decide to remain in the unit voluntarily……The compulsion and the nursing care should be so good that we would wish to put our own daughters here* (Gina).

However, nurses also recognised that the insertion of a tube under restraint represents the most invasive form of coercion.

In striving for a collaborative and supportive environment, nurses expressed a desire for NGT-FR to be administered in a manner encouraging patients’ active participation. Respecting patient autonomy was of the utmost importance, even during compulsory care, though it was challenging due to patients’ strong anxieties related to eating.

*When patients are too ill to eat on their own*,* it’s still essential to respect their autonomy. But it’s also a matter of care and respect for their lives to intervene and make sure they get the nourishment they need* (Lilly).

NGT-FR often started with a step-by-step strategy prioritising voluntary cooperation. The nurses made earnest efforts to engage patients voluntarily, only resorting to involving other personnel when all other possibilities had been explored. Thea articulated this process:

*First*,* we ask*,* then we try [to encourage them to eat]. Oh*,* we try and try. We try again*,* and then if it doesn’t work*,* we ask about their thoughts on the matter*,* if we should insert it [the nasogastric tube]. Typically*,* two people are involved where one assists… And if the situation doesn’t improve*,* then we have to call personnel [from another ward] to come and assist* (Thea).

Despite the necessity of NGT-FR, maintaining a balance between empathy and encouragement remained central to the nurses’ care philosophy. They believed in motivating patients, even in the face of initial resistance. NGT-FR was not solely viewed as a restrictive measure but as a chance to provide patients with a sense of control within the care plan. As Gina said: *For them*,* having some choices*,* even in compulsory treatment*,* is essential. It’s a form of good compulsory care or treatment*.

The nurses recognised the need for a balance between empathy and resolution. Some emphasised warm and empathetic encounters, others the necessity of assertiveness in situations requiring compulsory treatment. Some even stressed the importance of combining both approaches. Mia described this balance:

*It is communicated in the ward that we have to be a little careful not to get too warm and affectionate in the tube situations*,* because it can trigger a desire for empathy in those situations*,* and maybe make her not eat*,* but rather go all the way and be force fed. Because then she knows she’s getting some extra attention. You have to be warm and understanding*,* but you also have to be firm. This balance is how we optimise the effectiveness of treatment* (Mia).

Navigating the fine line between being a helper and exerting control over the patient was described as a dilemma (Beatrice). However, when NGT-FR was administered, it was generally conducted discreetly behind closed doors, aimed at maintaining patient confidentiality and minimising distress for both the patient in treatment and other patients on the ward. The closed-door approach ensured that the situation did not escalate into a public spectacle.

### Having ethical concerns about NGT-FR when the patient reaches a BMI not immediately life-threatening

Nurses reflected on the ethical implications of NGT-FR, especially in cases where a patient’s BMI reached a level not immediately life-threatening. Then, some nurses found it ethically questionable to continue NGT-FR. They expressed their concerns in ward meetings and questioned the appropriateness of the care they provided.

*I feel it’s challenging to be a nurse and insert a tube when their BMI is fifteen or maybe sixteen*,* but the situation around the patient has reached a point where a nutritional decision has been made to ensure they receive nourishment*,* and they should mostly eat on their own or take the supplement if they find food too difficult …these with a high BMI*,* in refusal to eat and tube feeding. It’s hard! ….* (Rosie).

*…If it’s not life-threatening*,* and we’re still doing tubes*,* then I can’t understand that it’s a good treatment. In my world*,* it’s not.* (Luke)

Evelyn described how she sometimes experienced NGT-FR as so ethically problematic that she refused to take part in it: she had to either withdraw from the patient’s care team or refuse to carry out the doctor’s prescription of the NGT-FR.

*We have a patient who reached a high BMI*,* and I think it [NGT-FR] has been unethical. And I’ve said several times in the team meetings*,* that I don’t think it’s okay what we do. So*,* the last time she was here … I said I’m going to refuse to do it … no matter what she (chief) says*,* I refuse to do it. And I put my foot down because I couldn’t defend what we were doing* (Evelyn).

### Having concerns about involving personnel from another ward in the NGT-FR procedure

Nurses struggled with the question of who should perform the NGT-FR procedure. The routine practice on the ward stipulated that when a patient needed NGT-FR, personnel from another ward were called on to assist in restraining the patient while the nurse inserted the tube and provided nutrition.

*When the patient doesn’t finish their meal*,* they are offered a drink*,* and if they refuse that*,* a formal decision is made. What happens then is that we call the [name of the ward]*,* and two strong men arrive to restrain the patient while we insert the feeding tube.* (Luke)

Certain situations presented explicit difficulties for nurses, particularly when patients showed strong resistance and were uncooperative. Some questioned the procedure of involving personnel from another ward to hold the patient, believing that it might be better if the nurses themselves conducted the procedure, considering the potential distress it could cause to patients who might have a history of abuse. *….I’ve thought that it might be best if I personally restrained the patient*…. (Thomas).

Some viewed the tubing procedure as an integral part of providing high-quality care for severely ill patients and as such did not experience moral conflicts associated with the use of restraint. They considered the intervention to be lifesaving.

*As a nurse*,* I don’t feel any internal conflict. When it becomes too challenging for the patient*,* we step in. I appreciate our ward’s closeness to the other ward and our dialogue. They’re trained to manage patients in various situations*,* allowing me to focus on inserting the tube and handling the situation*,* knowing that there are skilled individuals ensuring the patient’s comfort* (Rosie).

However, participants expressed concerns about the relationship with patients, emphasising that inserting a tube was not merely a technical task but also a relational one. They stressed the significance of fostering a strong bond and forming an alliance with the patient, and some believed that holding the patient during restraint could hinder the establishment of a meaningful and lasting relationship. Nancy told of an episode where she performed the procedure (tube insertion) on a patient without knowing the patient beforehand. As a result, they never developed a good relationship, even though the patient was admitted to the ward several times.

*I was put in a situation where I was going to the room and inserting a tube with a pump and starting the pump. What was unfortunate was that I wasn’t presented to the patient in advance. I was just going in and doing a job. I felt like that meeting ruined something….It has something to do with being a little prepared for the first meeting and maybe meeting them in a more respectful way* (Nancy).

After the tube insertion, nurses described how patients often felt regretful and frequently cried. Nurses who had witnessed the restraint found themselves in the role of providing comfort, a task that could be emotionally challenging, because they had also contributed to the procedure.

*Subsequently*,* the patient is often in a state of despair*,* crying and falling apart. You sit there*,* attempting to comfort her for perhaps an hour*,* helping her catch her breath and calm down. First*,* you’ve been a part of the procedure and afterward you need to comfort the patient. It’s as if the executioner is comforting the victim* (Luke).

## Discussion

The aim of our study was to gain a deeper understanding of nurses’ experience of NGT-FR in hospitalised patients with severe AN. Three main themes were developed: providing good nursing care during coercive treatment; having ethical concerns about NGT-FR when the patient reaches a BMI not immediately life-threatening and having concerns about involving personnel from another ward in the NGT-FR procedure.

This study focuses on the nurses’ experiences with the most severe cases of AN, with many patients in need of NGT-FR for survival. This intervention is critical for individuals who are unable to meet their nutritional needs due to the severity of their condition. The provision of NGT-FR can be a lifesaving measure, ensuring that patients receive the essential nutrients needed for recovery and maintenance of health. Nevertheless, NGT-FR probably represents the most invasive form of coercion for patients and raises many ethical and legal issues for nurses [4, 9–11]. The biomedical principles of beneficence, non-maleficence and autonomy seem relevant to this discussion [22]. Beneficence refers to the duty to do good for others, non-maleficence is the duty to avoid harming them, and autonomy entails a duty to respect individuals’ capacity for self-governance and their right to make choices [22]. The principle of patient autonomy has gained an increasingly prominent role in healthcare ethics and medical decision-making in our part of the world. However, in severe and life-threatening conditions such as patients with AN without competence to consent, there arises a critical need to assess whether the patient poses a danger to their life. Even though most patients lack competence to consent, nurses strove to maintain patients’ autonomy as far as possible during NGT-FR. In such circumstances, the principle of beneficence and non-maleficence must take precedence over autonomy [22].

Rendtorff and Kemp introduce the principles of dignity, integrity, and vulnerability, which play a pivotal role in healthcare [23, 24]. Dignity, as defined by Rendtorff: ‘expresses the intrinsic worth and equality of all human beings and expresses the moral responsibility of the human person’ [25 p. 117]. This principle holds particular significance for the care of individuals with life-threatening AN, emphasising the imperative need for treating them with respect and compassion. Nurses must endeavour to uphold the dignity of patients even during NGT-FR. In this study, nurses were concerned about letting patients decide as much as possible before, during and after NGT-FR. Nurses also made earnest efforts to engage patients voluntarily, as a way to uphold patients’ dignity. Integrity refers to the requirement that human life remain inviolate, its coherence being preserved, especially in the face of illness and vulnerability [23, 25]. Vulnerability, closely intertwined with integrity, points to the inherent fragility of the human condition, and its management is paramount in healthcare settings such as the care of the patients in our study: ’Medicine’s action directly concerns bodily vulnerability; the human person, however, is both object body and lived body` [25 p.118]. NGT-FR should therefore be conducted with sensitivity, recognising and respecting the patient’s vulnerability while striving to ensure their well-being.

**Fig. 1:**
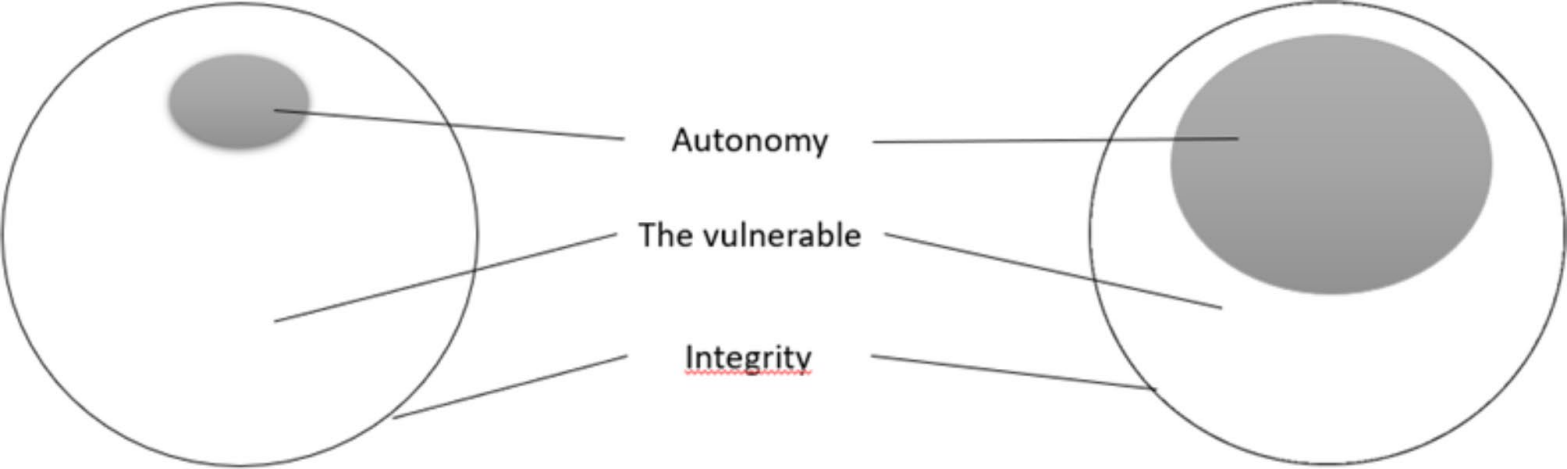
How autonomy and vulnerability can vary in a person’s life according to Braut [26]

Braut argues that people’s autonomy varies throughout life, depending on, for example, age and disease [26]. Although autonomy varies, human dignity and integrity remain constant. This means, for example, that if people are unable to make autonomous decisions, they are still entitled to have their physical and mental limits respected. Figure 1 shows how autonomy and vulnerability can vary in a person’s life. Situations where patients are highly vulnerable place greater demands on healthcare personnel to safeguard their autonomy and integrity [26].

The circle to the left may represent a patient with AN in need of NGT-FR, with a low degree of autonomy, and a high degree of vulnerability, but with equal integrity as in the circle to the right, (representing a healthy person with a high degree of autonomy). Patients with AN in need of NGT-FR are among the most vulnerable individuals [27]. At a low degree of autonomy, patients are even more vulnerable and dependent on others to maintain their dignity. Although autonomy is important, beneficence, non-maleficence, dignity, integrity, and vulnerability are perhaps equally important in discussing this issue.

In severe AN, NGT -FR is sometimes essential and lifesaving. Some nurses viewed the tubing procedure as an integral part of providing high-quality nursing care for severely ill patients and did not experience moral conflict associated with the use of restraint, considering the intervention to be lifesaving and in conformity with the legislation. This is in line with a meta-synthesis review of studies with nurses and care staff, showing that managing physical restraint feels unpleasant but necessary [3]. Most participants in the meta-synthesis [3] expressed concern about the relationships with patients, emphasising that inserting a tube was not merely a technical task but also relational one. In another study, nurses disclosed that they did not feel that physical restraint damaged the therapeutic relationship, but that the team’s support was important [6]. Nurses in our study found themselves balancing between non -maleficence and beneficence. In doing so, the nurses tried to safeguard patients` autonomy motivated patients to active participation and pursued a step-by -step strategy prioritising voluntary cooperation and gave patients choices about how the compulsory procedure should be performed. In a study of patients` views of good coercion [11], ensuring patient autonomy before and during the use of coercive measures, clear communication, and mutual understanding about why coercion is used, secure and trustworthy relations and being seen as a person were highlighted. This corresponds to the results of Tan et al.‘s study, which showed that patients found compulsory treatment and coercion appropriate when the condition was life -threatening [27]. What mattered most was not whether patients had experienced restriction of freedom or choice, but the nature of their relationship with mental health professionals. Good nursing during NGT-FR means balancing empathy, encouragement, and resolution. Nurses in our study described navigating the line between being a helper and exerting control over the patient as a dilemma. This is in line with research pointing to the importance of maintaining dignity and vulnerability in patients` lives [28–30].

The nurses described situations where they experienced unnecessary and unethical NGT-FR. They were concerned about the rigidity of rules and regulations, the need for individualisation, and the importance of discretion when conducting NGT-FR. Many reflected on the ethical dilemmas of tube feeding, especially in cases where a patient’s BMI reached a level not immediately life-threatening. In situations where coercion is perceived as necessary and ethically justifiable, NGT-FR seems necessary to save lives. Once patients have reached a higher BMI, and are close to discharge from the hospital, this is no longer the case. In these situations, nurses might think that NGT-FR is not in line with the principle of beneficence [22], that treatment under restraint harms more than helps. The reason why coercive decisions are sometimes upheld even though the situation is no longer life-threatening is that some patients with low BMI may not have decision-making abilities or mental capacity to consent [30, 31]. These individuals might cease nutritional intake if given autonomy, potentially leading to rapid weight loss and return to life-threatening conditions. Consequently, any prior treatment and resources expended would be wasted. Thus, coercive measures align with Norwegian legislation (“an assessed incapacity of the patient to consent”) [9, 10] and are considered ethically justifiable to ensure the treatment is seen through completion. Still, this approach has led some nurses to opt out due to ethical concerns.

If we return to Fig. 1, the circle to the left may represent a patient in need of NGT-FR, with low autonomy and high vulnerability, needing nurses to protect her integrity. The circle to the right may represent a patient who is no longer dependent on NGT-FR, with higher autonomy, but still vulnerable. However, it may also represent the nurse, also vulnerable, and in need of protecting her human and professional integrity. Even if NGT-FR is necessary and lifesaving, our findings indicate that the nurses found it difficult and demanding to be in charge of coercion, (being both “helper and executioner”), and participating in treatment seeming to violate the patients’ vulnerability and integrity [13, 28]. Nurses are also vulnerable and being “the executor” might also challenge nurses` integrity. This is in line with Kodua et al. s’ s study of nursing assistants showing that they experienced emotional distress, physical exhaustion, pressure and responsibility related to NGT- FR [7].

Our last finding relates to the question of involving personnel from another ward for the NGT-FR procedure. Once it has been decided that NGT-FR must be carried out, is it preferable to enlist additional nurses from a different ward to assist in holding the patient during the procedure or might this be perceived as even more traumatising for patients, and especially for some with a history of abuse? The nurses did not reach a consensus regarding this. The nurse Thomas in the introductory narrative reflects on whether the holistic care and nursing (which here also includes NGT-FR) can be perceived as optimal for the patient if the same nurse is involved all the way, and also holds the patient during NGT-FR. Other nurses stressed the significance of fostering a strong bond and forming an alliance with the patient, believing that holding the patient during restraint could hinder the establishment of a meaningful and lasting relationship. The participants said that it was frustrating to be both “helper and an executioner”. This echoes the findings from Bommen et al. [13]. Further studies with nurses, and patients with AN, are needed to investigate whether it is best for the patient and the therapeutic relationship, with unknown caregivers restraining the patient during NGT-FR.

### Study limitations

The participants predominantly consisted of woman nurses with significant work experience in one specific ward, which may have influenced the interview outcomes. A more diverse group, including more men nurses and less experienced nurses, as well as nurses from other hospitals, could potentially have added greater depth to the findings. The study was conducted by authors who are all registered nurses with diverse clinical experience, although they did not have experience in this specific field. Despite this, the interviewer possessed extensive research experience with the patient group in question. This dual professional identity bridges healthcare and research but also presents challenges for nurses, warranting greater attention [32]. While the absence of clinical experience in AN of all authors could be seen as a limitation, conversely, it can also be argued to be a strength as it can provide a more curious open-minded approach allowing the interviewer to ask questions that clinicians might expect an insider to know. The data-driven approach and reflexivity throughout the study helped mitigating these limitations [18, 19]. The research team actively engaged in discussions during the analysis to maintain awareness of preconceptions, ensuring that all authors were involved in every aspect of the study.

## Conclusions

Our study reveals that nurses find NGT-FR to be part of life-saving nursing in patients with severe AN. According to Norwegian legislation all coercive measures, such as NGT-FR must be legally justified. Still, NGT-FR raises ethical concerns, especially with patients with a BMI no longer life-threatening. Nurses are also concerned about who should perform the restraint. Our findings show that nurses are also vulnerable, and that nursing during NGT-FR is demanding and ethically challenging.

## Data Availability

Due to privacy and ethical restrictions, data are not publicly available. Anonymous data that support the findings are available upon reasonable request to the authors.

## Abbreviations

AN: Anorexia Nervosa
BMI: Body Mass Index
DSM-V: Diagnostic and Statistical Manual of Mental Disorders, Version Five
NGT-FR: Nasogastric Tube Feeding Under Restraint
